# Cell-Adhesive Bioinspired and Catechol-Based Multilayer Freestanding Membranes for Bone Tissue Engineering

**DOI:** 10.3390/biomimetics2040019

**Published:** 2017-10-05

**Authors:** Maria P. Sousa, João F. Mano

**Affiliations:** CICECO—Aveiro Institute of Materials, Department of Chemistry, University of Aveiro, 3810-193 Aveiro, Portugal; mariajsousa@ua.pt

**Keywords:** mussel-inspired, biomimetic, dopamine, multilayer freestanding membranes, adhesiveness, osteogenic differentiation, bone tissue engineering

## Abstract

Mussels are marine organisms that have been mimicked due to their exceptional adhesive properties to all kind of surfaces, including rocks, under wet conditions. The proteins present on the mussel’s foot contain 3,4-dihydroxy-l-alanine (DOPA), an amino acid from the catechol family that has been reported by their adhesive character. Therefore, we synthesized a mussel-inspired conjugated polymer, modifying the backbone of hyaluronic acid with dopamine by carbodiimide chemistry. Ultraviolet–visible (UV–Vis) spectroscopy and nuclear magnetic resonance (NMR) techniques confirmed the success of this modification. Different techniques have been reported to produce two-dimensional (2D) or three-dimensional (3D) systems capable to support cells and tissue regeneration; among others, multilayer systems allow the construction of hierarchical structures from nano- to macroscales. In this study, the layer-by-layer (LbL) technique was used to produce freestanding multilayer membranes made uniquely of chitosan and dopamine-modified hyaluronic acid (HA-DN). The electrostatic interactions were found to be the main forces involved in the film construction. The surface morphology, chemistry, and mechanical properties of the freestanding membranes were characterized, confirming the enhancement of the adhesive properties in the presence of HA-DN. The MC3T3-E1 cell line was cultured on the surface of the membranes, demonstrating the potential of these freestanding multilayer systems to be used for bone tissue engineering.

## 1. Introduction

Catechol-based materials have been investigated quite a bit in the last decade, presenting an interesting structural and chemical versatility capable of being applied in different fields such as food and agrochemical engineering, green technology, analytical, materials science, biomedicine, and biotechnology [1,2,3]. In fact, catechols are aromatic derivatives with two contiguous (ortho-)hydroxyl groups that occur ubiquitously in nature, being a part of different biochemical processes and functions as simple molecular units or even as macromolecules [4,5]. These interesting molecules occur in different systems such as fruits, tea, and insects, but it is on marine mussels that catechols were identified as being responsible for their extremely adhesive properties under wet conditions [4,6]. This adhesiveness is mediated by a class of protein, the mussel adhesive proteins (MAPs), known for containing a high amount of the noncationic amino acid 3,4-dihydroxy-l-alanine (DOPA) [7,8]. Intense research has been conducted in this field over the last few decades and it has been proven that the catechol element of DOPA is mainly responsible for the strong adhesion to different type of wet substrates, from inorganic to organic ones [7,9]. To develop MAPs-based materials, different strategies have been employed [10]. Recombinant DNA technology permits the engineering of MAPs precursors purified from *Escherichia coli* and converting into DOPA-containing mimetic by tyrosinase treatment [11]. Another strategy is the chemical modification of polymer backbones with adhesive moieties; for instance, DOPA-modified poly(ethylene glycol) macromers were synthetized through standard peptide chemistry and suggested to produce catechol-based hydrogels [12]. Also DOPA-modified poly(vinyl alcohol) hydrogels were produced with self-healing and pH-responsiveness properties [13]. The chemical modification with DOPA or its derivatives (like dopamine (DN)) of different natural polymers has also been investigated in recent years, from chitosan [14] to alginate [15], or even dextran [16] or hyaluronic acid [17]. For instance, DOPA-modified alginates with different substitution degrees were synthesized and used to produce membranes with enhanced adhesive and biocompatible properties [18].

Natural-based polymers are generally recognized by their high biocompatibility when compared with synthetic ones, exhibiting some recognition domains for cell-binding or cell-mediated processes and making them very interesting materials for tissue engineering and biomedical applications [19]. However, some challenges remain; mechanical properties and controlled biodegradability have been gaining increasing importance. Therefore, some strategies have been envisaged to enhance the potential of tissue engineering and biomedical products and their bioactivity, modifying the physico-chemistry or even the topography of the materials [20,21]. To improve the adhesive properties of materials, mainly in a hydrated environment such as the human body, researchers have been investigating the marine mussel system and taking advantage of the chemical reactivity of catechol moieties [6,22,23].

In this study, a biomimetic approach was combined with the use of natural-based polymers to obtain a mussel-bioinspired system for tissue engineering purposes, focusing on bone tissue engineering. Nowadays, autologous or allogeneic bone transplantation are still the most employed strategies for bone defect treatment, but it entails some risks of causing secondary trauma or even immune system rejection [24]. To overcome these drawbacks, several efforts have been applied to find a bone substitute that is ideally composed of three elements: cells, support material, and bioactive agents. An ideal tissue substitute must mimic the extracellular matrix (ECM) and different material parameters should be taken into account, such as the chemical groups, the biochemical properties, and the topography at the material–cell interface [25,26]. To date, different processing methodologies have been suggested to produce bone substitutes where the support material mimics ECM; nano- and microscale control of different properties along with the deposition of hierarchical films have been suggested for this purpose [27,28].

Layer-by-layer (LbL) is a versatile and inexpensive technique that has been widely applied in this context, being based on the sequential of complementary multivalent molecules on a substrate via electrostatic and/or nonelectrostatic interactions [29,30,31,32]. Different authors have reported LbL strategies to mimic some aspect of ECM [33]; Mhanna et al. [34] coated polydimethylsiloxane substrates with specific ECM macromolecules using LbL technology; they used collagen type I, chondroitin sulfate, and heparin and, depending on the composition of the film, they studied specific ECM–cell interactions. Layer-by-Layer-based products have also been exploited for bone tissue engineering purposes, offering fine control over different parameters like film thickness, architecture, chemistry, and even mechanical and topographical properties [35,36]. Oliveira et al. [37] assembled 10 tetralayers of human platelet lysates and marine-origin polysaccharides by LbL technology and then shaped them into fibrils by freeze-drying; the resulting scaffolds could induce the differentiation of human adipose stem cells into mature osteoblasts. In turn, Crouzier et al. [38] showed that cross-linked poly(l-lysine)/hyaluronic acid (HA) can serve as a reservoir for recombinant human bone morphogenetic protein-2 (rhBMP-2) delivery to myoblasts and induce their differentiation into osteoblasts in a dose-dependent manner. Overall, the LbL technique allows us to produce bioinspired and tunable materials to local deliver immobilized growth factors or other bioactive agents and even to instruct stem cells towards osteogenic phenotypes. Moreover, these films can be deposited on an extensive range of substrates of different composition, size, and shape.

Herein, LbL methodology was used to produce bioinspired freestanding multilayer membranes containing catechol groups on the surface and the bulk to improve the adhesiveness properties of the material. In this sense, DN moieties were chemically grafted onto HA to develop multilayer membranes through electrostatic interactions with chitosan (CHT). The modification of HA was confirmed by nuclear magnetic resonance (NMR) and ultraviolet–visible (UV–Vis) spectroscopy. The ability to construct multilayer films was monitored using quartz crystal microbalance with dissipation (QCM-D). Adhesion mechanical tests and in vitro adhesion assays assessed the effect of having DN on the performance of the freestanding multilayer membranes.

Overall, the combination of the versatility of LbL methodology with the protein mussels’ inspiration prompted us to exploit catechol-containing multilayer membranes to enhance the interfacial interaction between cells and materials, which takes advantage of the adhesive properties conferred by DN moieties. The potential to induce in vitro bone tissue regeneration was investigated, using a pre-differentiated MC3T3-E1 cell line.

## 2. Materials and Methods

### 2.1. Materials

Chitosan with a *N*-deacetylation degree of 80% and a molecular weight in the range of 190–310 kDa, HA as hyaluronic acid sodium salt from *Streptococcus equi* with a molecular weight in the range of 1500–1800 kDa, DN as dopamine hydrochloride with a molecular weight of 189.64 g mol^−1^ and *N*-(3-dimethylaminopropyl)-*N*′-ethylcarbodiimide hydrochloride (EDC) (purum ≥ 98.0% (AT)) were purchased from Sigma (St. Louis, MO, USA). These materials were used as received, except CHT, which was purified afterwards, following a standard procedure reported elsewhere [39].

### 2.2. Synthesis of Dopamine-Modified Hyaluronic Acid

The conjugate of HA modified with DN was synthesized using EDC as an activation agent of the carboxyl groups on HA chains, based on the procedure proposed by Lee et al. [40]. Basically, 1 g of HA was dissolved in 100 mL of phosphate-buffered saline (PBS, Sigma) solution and the pH was adjusted to 5.5 with a hydrochloric acid (HCl, 37%, reagent grade, Sigma) aqueous solution. Then, this solution was purged with nitrogen for 30 min and mixed with EDC and DN and maintained in reaction at 4 °C for 3 h. The pH was maintained at 5.5. Extensive dialysis was performed to remove unreacted chemicals and urea byproducts. After this step, the resulting conjugate was lyophilized for one week and then stored at −20 °C, protected from the light, to avoid oxidation.

#### 2.2.1. Ultraviolet–Visible Spectrophotometry

Ultraviolet–visible spectrophotometer (Jasco V-560 PC) was used to confirm the substitution of dopamine in the conjugate. A solution of 1 mg mL^−1^ in sodium acetate buffer (Scharlab, Barcelona, Spain) with 0.15 M sodium chloride (NaCl, LabChem, Pittsburgh, PA, USA) at pH 5.5 was prepared for the UV–Vis analysis and placed in 1 cm quartz cells. The wavelength range used for this analysis was from 190 nm to 900 nm. Sodium acetate buffer with 0.15 M sodium chloride and at pH 5.5 was used as the reference solution.

#### 2.2.2. Nuclear Magnetic Resonance

^1^H-NMR analyses were made dissolving overnight the HA, DN and HA-DN in deuterated water (D_2_O, Cambridge Isotope Laboratories, Inc., Andover, MA, USA) at a concentration of 1 mg mL^−1^ (^1^H-NMR). The spectra were obtained using a spectrometer BioSpin 300 MHz (Bruker, Billerica, MA, USA). The spectra were recorded at 298 K and 300 MHz for ^1^H.

### 2.3. Quartz Crystal Microbalance with Dissipation

The formation of the multilayers of CHT and HA-DN was followed in situ by QCM-D (Q-Sense, Biolin Scientific, Göteborg, Sweden). The mass change results from the variation of the normalized resonant frequency (Δ*f*/υ) of an oscillating quartz crystal when adsorption occurs on the surface and the dissipation factor (Δ*D*) provides a measure of the energy loss in the system. The measurements can be conducted at the fundamental frequency and at several overtones number (υ = 1, 3, 5, …, 11). Chitosan was used as the polycation while HA or HA-DN acted as the polyanion. Fresh polyelectrolyte solutions were prepared by dissolution of HA-DN, HA, and CHT in sodium acetate buffer containing 0.15 M of NaCl to yield a final concentration of 1 mg mL^−1^, at pH 5.5. The sensor crystals used were AT-cut quartz (Q-Sense) with gold-plated polished electrodes. These crystals were excited at 5 MHz as well as at 15, 25, 35, 45, and 55 MHz corresponding to the 3rd, 5th, 7th, 9th, and 11th overtones. The crystals were previously cleaned with a pre-exposition to UV ozone (BioForce Nanosciences, Salt Lake City, UT, USA) irradiation during 10 min followed by an immersion on a 5:1:1-mixture of mQ-water, ammonia (25%, Sigma), and hydrogen peroxide (30%, Sigma) at 75 °C, during 5 min. Then the crystals were exposed to a sequential sonication for 3 min in acetone, ethanol, and isopropanol (all from Sigma) and then dried with flowing nitrogen gas avoiding contamination prior to use. To ensure that the crystals are perfectly clean and therefore show a null frequency, all the experiments started with a buffer/solvent baseline. Then, the polyelectrolyte solutions were injected into the cell during 10 min at a flow rate of 20 μL min^−1^, starting with CHT. A rinsing step of 10 min with the solvent was included between the adsorptions of each polyelectrolyte. The multilayer systems were assembled at pH 5.5. The pH was adjusted with HCl or sodium hydroxide (NaOH, pellets, Fine Chemicals, Akzo Nobel Chemicals S.A., Mons, Belgium). Chitosan/HA films were prepared for comparison, to conclude about the DN effect onto the multilayer system. Films with eight bilayers were produced. All experiments were conducted at 25 °C. During the entire process Δ*f*/υ and Δ*D* shifts were continuously recorded as a function of time. Thickness measurements were performed using the Voigt viscoelastic model implemented in the QTools software (Q-Sense, version 3.1.29.619). Changes in resonant frequency and dissipation of the fifth overtone were fitted. Based on the assumed growth models, the thickness of the multilayer films after 200 cycles was estimated for each system.

### 2.4. Freestanding Production and Characterization

Multilayer CHT/HA and CHT/HA-DN were built on polypropylene (PP) substrates (Auchan, Villeneuve-d’Ascq, France). Prior to the depositions, these surfaces were cleaned with ethanol and rinsed thoroughly with water before being dried with a stream of nitrogen. The polyelectrolyte solutions were freshly prepared at 1 mg mL^−1^ in a sodium acetate solution containing 0.15 M NaCl, being the pH adjusted to 5.5. The PP substrates were firstly dipped in CHT solution for 6 min and then rinsed twice in the washing solution (acetate buffer, pH 5.5) for 2 min each. Then, they were immersed in the polyanion solution (HA or HA-DN) for 6 min and again twice in the washing solution. This procedure was repeated 200 times with the help of a homemade dipping robot. After drying, the multilayer films were easily detached from the PP substrates without any damage resulting on the freestanding membranes [CHT/HA]_200_ and [CHT/HA-DN]_200_. After production, the membranes were characterized using different techniques and equipment.

#### 2.4.1. Scanning Electron Microscopy and Energy-Dispersive X-ray Spectroscopy

The surface morphology of both sides of [CHT/HA]_200_ and [CHT/HA-DN]_200_ membranes was observed using a Hitachi SU-70 (Hitachi, Tokyo, Japan) scanning electron microscope. All samples were coated with a conductive layer of sputtered gold/palladium. The scanning electron microscopy (SEM) micrographs were taken at an accelerating voltage of 4 kV and at different magnifications. For the cross-section observation, the detached freestandings were immersed in liquid nitrogen until free fracture. After that, the free fracture was placed at 45° and observed by SEM. Energy-dispersive X-ray spectroscopy (EDS, Hitachi) was also used to determine the elemental components of the top surface and in the cross-section of the membranes. The samples were also sputtered with gold/palladium and the analysis was made at an accelerating voltage of 15 kV. The ratio between the oxygen (O) and the nitrogen (N) presented on the top surfaces was quantified in a representative area of the membrane (A = 0.136 mm^2^).

#### 2.4.2. Adhesive Mechanical Tests

The adhesion properties of the multilayer were firstly evaluated using a universal mechanical testing machine (Instron model 5966, High Wycombe, Buckinghamshire, UK), following the standard test method for shear strength of single-lap-joint adhesively bonded metal specimens by tension loading ASTM D1002 (ASTM International, West Conshohocken, PA, USA) with slightly modifications. All the adhesion experiments were conducted at 25 °C, at a cross-head speed of 5 mm min^−1^ and using a 1.0 kN static load cell. The lap shear adhesion specimens were squares (20 mm × 20 mm) of freestanding membranes that were incubated at 37 °C and equilibrated in a 50% humidity atmosphere prior to testing. Briefly, the samples were put between two glass slides and left in contact overnight. Then, the systems were stressed until enough force was applied to trigger their detachment and pull them apart, using the Instron apparatus. The lap shear bonding strength was then determined from the maximum of the force–deformation curve obtained. The average and standard deviations were determined using the results from five samples.

While lap shear strength gives a quantitative idea of the adhesive properties of the membranes, other nonconventional test was made to observe the bioadhesiveness potential of these systems. Briefly, the [CHT/HA]_200_ and [CHT/HA-DN]_200_ freestanding membranes were put in contact with a clean surface of porcine bone tissue in a 50% humidity controlled environment at 37 °C. Then, the freestanding membranes were pulled out of the bone with tweezers. This process was recorded by a video camera (Canon EOS 1200D, Tokyo, Japan).

### 2.5. In Vitro Cellular Tests

The sub-clone 4 of MC3T3-E1 cell line was obtained from the American Type Culture Collection (ATCC)-Laboratory of the Government Chemist (LGC) standards (ATCC^®^ CRL-2593™) [41]. The cells were cultured with Minimum Essential Medium (MEM) Alpha Modification (1X), (α-MEM, Gibco, Thermo Fisher Scientific, Waltham, MA, USA) supplemented with sodium bicarbonate suitable for cell culture (Sigma), 10% fetal bovine serum (FBS, Life Technologies™, Thermo Fisher Scientific, Waltham, MA, USA), and 1% antibiotic–antimycotic (Gibco) at pH 7.4. The cells were cultured in 75 cm^2^ tissue culture flasks and incubated at 37 °C in a humidified air atmosphere of 5% CO_2_. The medium was changed every three–four days. At 80–90% of confluence, cells grown in tissue culture flasks were washed with Dulbecco’s phosphate-buffered saline (DPBS, Corning, NY, USA) and then detached by a chemical procedure with trypLE™ express solution (Life Technologies™) for 5 min at 37 °C in a humidified air atmosphere of 5% CO_2_. To inactivate the trypLE™ express effect, cell culture medium was added. The cells were then centrifuged at 300 × *g* and 25 °C for 5 min and the medium was decanted. Cells between passage 12 and 13 were used for this study. Prior to cell seeding, the samples were cut in small squares of 25 mm^2^ or 1 cm^2^, treated with UV ozone for 10 min and immersed in ethanol for 2 h. Then, 150 μL or 300 μL (depending on the size of the sample) of supplemented α-MEM containing a cell suspension with a density of 2 × 10^4^ cells cm^−2^ was dropped above the surfaces of the [CHT/HA]_200_ and the [CHT/HA-DN]_200_ freestanding membranes, and the positive control tissue culture polystyrene surface (TCPS, Sarstedt AG & Co., Nümbrecht, Germany) (in triplicate). Then, the samples were incubated at 37 °C in a humidified air atmosphere of 5% CO_2_. After 4 h, cells already started to adhere and fresh basal culture medium was added.

#### 2.5.1. Metabolic Activity of MC3T3-E1 Cells

The samples were tested for cytotoxicity by analyzing their metabolic activity using the alamarBlue^®^ reduction assay (Invitrogen™, Thermo Fisher Scientific, Waltham, MA, USA). Briefly, the samples (small squares of 25 mm^2^) with adhered cells were placed in a nontreated surface 48-well cell culture plate (in triplicate) and incubated at 37 °C and 5% CO_2_. At one, three and seven days of culture, the assay was performed, always protecting from light. Briefly, the culture medium was removed and 500 μL of supplemented α-MEM containing 10% (*v*/*v*) of alamarBlue solution was added to each well. The samples were then incubated in the dark, overnight, at 37 °C and 5% CO_2_. After 12 h, 100 μL of each well (in triplicate) was transferred to a 96-well plate. The absorbance was monitored at 570 nm and 600 nm, using a microplate reader Synergy HTX (BioTek Instruments, Inc., Winooski, VT, USA).

#### 2.5.2. DNA Quantification Assay

A DNA quantification assay (Quant-iT™ PicoGreen^®^ dsDNA Assay Kit, Invitrogen™, Thermo Fisher Scientific) was also performed to evaluate cell proliferation when cultured on the samples’ surface. All seeding procedure was repeated for this assay. For each culture time, the samples were washed with DPBS, and then, transferred with 1 mL of ultrapure sterile water to an Eppendorf flask. These Eppendorf flasks were placed at 37 °C for 1 h and then immediately stored at −80 °C until use. The quantification of total DNA was determined after cell lysis, according with the manufacturer’s description. After transferring each solution to a 96-well white opaque plate (in triplicate), the plate was incubated at 25 °C, protected from the light, for 10 min. The standard curve for DNA analysis was generated with provided DNA from the assay kit. Fluorescence was read at excitation of 485/20 nm and emission of 528/20 nm using a microplate reader Synergy HTX (BioTek Instruments, Inc.).

#### 2.5.3. Morphological Observation of MC3T3-E1 Cells

MC3T3-E1 cell morphology was observed using a fluorescence microscope (Axio Imager 2, Zeiss, Oberkochen, Germany). Briefly, the cells were seeded above the samples (squares 1 cm^2^) at a density of 2 × 10^4^ cells cm^−2^ and cultured for three and seven days, using basal culture conditions. After each time-point, the samples were gently washed with sterile DPBS and fixed with 10% (*v*/*v*) of formalin (Sigma) in DPBS solution for 30 min. To obtain morphological fluorescence images, a rhodamine phalloidin (Thermo Fisher Scientific) and 4′,6-diamidino-2-phenylindole (DAPI, Thermo Fisher Scientific) fluorescent assay was performed at each time culture period; DAPI stains preferentially nuclei and phalloidin the actin fibers of the cell cytoskeleton. Firstly, the fixed samples were permeabilized with 0.2% (*v*/*v*) of Triton X-100 (Sigma) in DPBS solution for 10 min and then blocked with 5% FBS (*v*/*v*) in DPBS solution for 30 min. Then, the samples were treated with rhodamine phalloidin for 45 min and consequently with DAPI for 15 min. Afterwards, the cell morphology was observed using the fluorescence microscope.

#### 2.5.4. Osteogenic Potential of Dopamine-Modified Hyaluronic Acid Membranes and Differentiation of MC3T3-E1 Cells by Immunocytochemistry

To evaluate the osteogenic potential of these substrates, cells were cultured at 2 × 10^4^ cells cm^−2^ in basal growth medium, at 37 °C and 5% of CO_2_. After five days in basal conditions, the medium was changed for osteogenic medium (α-MEM containing 10% FBS, 10 mM β-glycerolphosphate disodium salt hydrate (Sigma), and 50 μg mL^−1^ l-ascorbic acid (Cayman Chemical, Ann Arbor, MI, USA)). The differentiation medium was changed every three days.

Intracellular osteopontin expression has been reported as a marker for osteogenic differentiation [42]. After 14 days in differentiation medium, the samples were fixed in 10% (*v*/*v*) formalin (Sigma) in DPBS. Following the fixation step, the fixed samples were permeabilized with 0.2% (*v*/*v*) of Triton X-100 in DPBS solution for 10 min and then blocked with 5% FBS (*v*/*v*) in DPBS solution for 60 min. Then, the samples were examined for protein expression visualization using a mouse antibody against osteopontin (BioLegend, San Diego, CA, USA), by incubation overnight, at 4 °C. Subsequently, the samples were treated with the corresponding secondary antibody anti-mouse Alexa Fluor 647 (Invitrogen™) for 1 h at 25 °C and consequently with rhodamine phalloidin and DAPI for 45 min and 15 min, respectively. Between each step the samples were extensively washed. Afterwards, the cell morphology was observed using fluorescence microscopy.

### 2.6. Statistical Analysis

All the experiments were performed with at least three replicates and were independently performed three times. Results are expressed as mean ± standard deviation. Differences between the experimental results were analyzed using the one-factor or two-factor analysis of variance (ANOVA), with the Bonferroni’s multiple comparison test, defined with a statistical significance of *p* < 0.05.

## 3. Results

**Figure 1:**
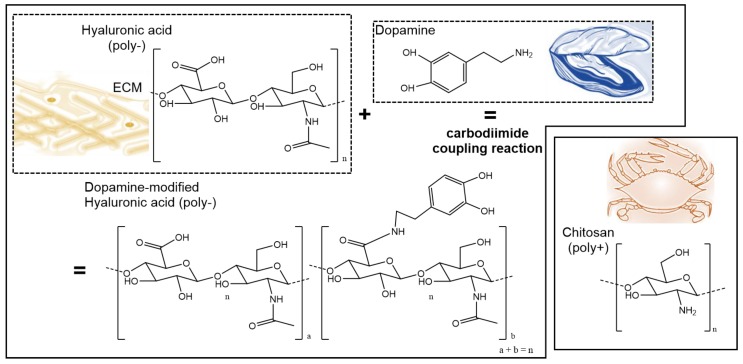
Chemical structure of hyaluronic acid (HA), dopamine (DN), and chitosan (CHT). Synthesis and chemical structure of dopamine-modified hyaluronic acid (HA-DN). ECM: extracellular matrix.

The HA was functionalized with DN and the resulting conjugate was combined with CHT to produce multilayer biomimetic membranes by LbL. The chemical structures of each compound, including the resulting chemical structure of HA-DN are represented in Figure 1. We hypothesize that the presence of DN moieties along the thickness of the films, especially on the last layer and top surface of the membrane, could enhance the interaction between cell and material and improve the osteogenic potential of MC3T3-E1 cells.

### 3.1. Synthesis and Characterization of Conjugated Dopamine-Modified Hyaluronic Acid

The conjugation of DN on HA backbone was achieved by the standard carbodiimide coupling method. Using EDC chemistry, the carboxyl group of HA was activated to react with the amine group of DN. After lyophilizing, the resulting conjugated was characterized by UV–Vis and NMR for ^1^H.

**Figure 2:**
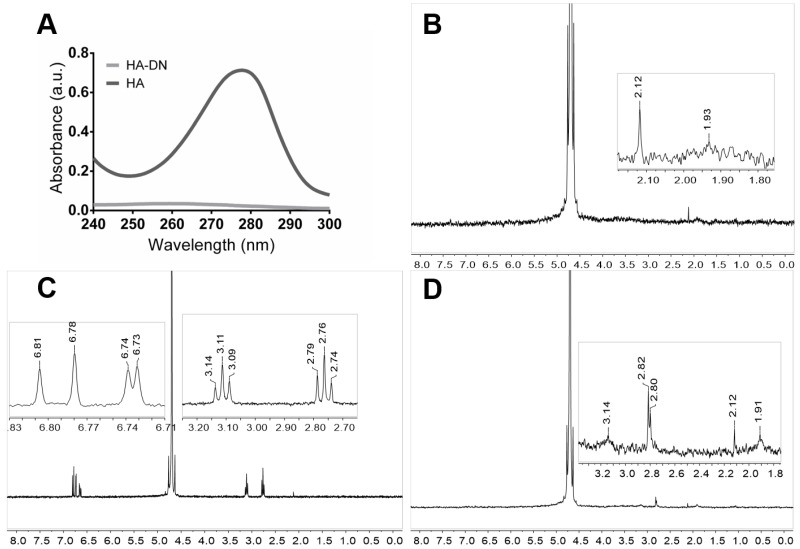
Characterization of conjugated dopamine-modified hyaluronic acid. (**A**) Ultraviolet–visible (UV–Vis) spectra of the control (HA) and the catechol-based conjugate (HA-DN). ^1^H-nuclear magnetic resonance (NMR) spectra of (**B**) HA; (**C**) DN and (**D**) the synthesized conjugate HA-DN, all with an expanded view. a.u.: Arbitrary units.

Figure 2A shows the UV–Vis spectrum of each polymer solution, HA and HA-DN. The conjugation of DN onto the backbone of HA was confirmed by the presence of a typical peak around 280 nm, characteristic of dopamine. As expected, this peak did not appear for HA, confirming the presence of DN moieties on the final conjugated HA-DN [43].

Figure 2B–D shows the NMR spectra of HA, DN, and HA-DN for ^1^H. Regarding the spectrum of HA, the peak at δ = 1.93 ppm is associated with the protons of the methyl group [44]. The spectrum of DN was characterized by the triplets centered at δ = 2.76 ppm and at δ = 3.11 ppm that are associated with the protons of the aliphatic group [45]. In turn, the multiplets between δ = 6.73 ppm and δ = 6.81 ppm are related with the protons in ortho- and meta-coupling position of the ring [17]. The spectrum of HA-DN was consistent with the HA and DN ^1^H-NMR spectra, as observed in Figure 2D. Both the results of UV–Vis spectroscopy and NMR confirmed that DN was successfully conjugated to HA.

### 3.2. Multilayer Construction and Thickness Estimation

**Figure 3:**
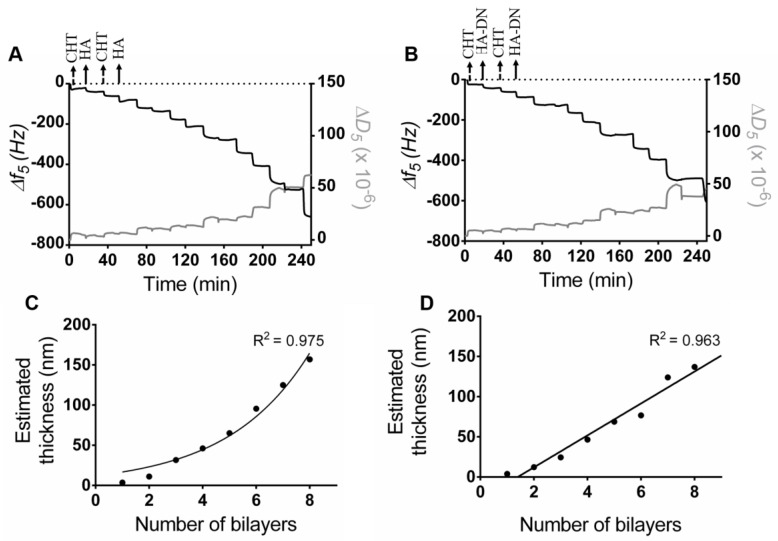
Build-up assemblies of (**A**) CHT and HA, and (**B**) CHT and HA-DN, monitored by quartz crystal microbalance with dissipation (QCM-D). Data shows the normalized frequency (Δ*f*) and dissipation (Δ*D*) variations at the fifth overtone as a function of the time. Cumulative thickness evolution of the (**C**) CHT/HA and (**D**) CHT/HA-DN multilayer systems as a function of the number of deposition bilayers (Voigt model).

The assembly of CHT and HA or HA-DN was first monitored on a gold quartz crystal by QCM-D. Figure 3A shows the LbL assembly of CHT and HA and Figure 3B shows the assembly of CHT and HA-DN. For both conditions, the first change in frequency happened when CHT was deposited on bare gold quartz. The second decrease in frequency corresponded to HA or HA-DN layers and this behavior was repeated during all the experiment (eight bilayers), indicating the successful of alternate adsorption of CHT and HA or HA-DN onto the quartz crystal surface. For both systems, the film seemed to have a stable growth for the first layers but it appeared more unstable with the addition of further layers. We hypothesize that it could be due to the formation of soluble macromolecular complexes between the previous layer and the new polymer solution [46]. Besides monitoring frequency variation (Δ*f*), QCM-D technology also detects dissipation variation (Δ*D*), allowing to take assumptions of the multilayer hydration state. When the film is rigid, the Δ*f* and Δ*D* for the fundamental frequency superpose with the signals recorded in the higher harmonic [47]; in the case of CHT/HA and CHT/HA-DN systems the soft nature of the layers adhering on the crystal leads to the dispersion of the different overtones. A more swollen multilayer film was achieved for polysaccharide-based films when compared with other LbL systems [48]. The assembly of CHT and HA or HA-DN induced similar frequency changes but different dissipation variations, with a bigger dissipation shift when CHT was deposited. This could be related to the fact that more water molecules were entrapped in the CHT layer [49].

Using the modeling tool Q-Tools [49], other parameters could be estimated like the thickness of the films. The Voigt model was chosen to estimate the film thickness of each system, requiring three parameters to be fixed: solvent density, solvent viscosity, and layer density. The solvent viscosity was fixed at 1 mPa s (the same as water) and the film density at 1100 kg m^−3^ (frequently assumed to return the lowest calculation error). The solvent density was changed by trial and error between 1000 and 1080 kg m^−3^ until error was minimized. Figure 3C,D represents the thickness estimation along with the number of bilayers for CHT/HA and CHT/HA-DN systems, respectively. For the CHT/HA system, the thickness of the films seemed to increase exponentially with the number of bilayers. On the other hand, CHT/HA-DN seemed to increase linearly with the number of bilayers. These results are in accordance with the related literature [17]; CHT/HA multilayer films are uniquely composed of polysaccharides and naturally more water-rich than the other system. In turn, the presence of DN on the CHT/HA-DN system seemed to change the growth regime to a linear model; this could indicate that DN conferred less water content on the films. After eight bilayers, the estimated thickness for the CHT/HA system is 157 nm and for the CHT/HA-DN it is 137 nm. Using the resulting linear model, after 200 bilayers we expected a thickness of the CHT/HA-DN system of about 4.0 µm. As HA-DN presents bigger chains, we expected that the thickness of the CHT/HA-DN could be higher than for the CHT/HA system. However, for the first eight bilayers, we observed the opposite trend. We hypothesize that it could be the result of the re-arrangement of the polymer chains when LbL happens; the HA-DN chains seemed to compact more than HA chains, for the first layers of the film.

### 3.3. Production of the Freestanding Multilayer Membranes

From QCM-D monitoring results, CHT and HA or CHT and HA-DN could be combined to produce multilayer systems, with times of deposition of about 10 min; although from QCM-D data, using 6 min of deposition would be enough to construct such multilayer films. This observation was valuable to reduce the times of processing. Therefore, to produce freestanding polyelectrolyte multilayered membranes, we dipped an inert substrate successively on CHT and HA or on CHT and HA-DN solutions, for 6 min each immersion. The chosen underlying substrates were simple PP sheets that are widely available, inexpensive, and can be cut into a large range of shapes and sizes [50]. Between each dipping, we realized a washing step in the sodium acetate buffer (pH 5.5), to remove the excess of polymer. After 200 bilayers, the resulting multilayer films were dried at 25 °C and then easily detached from the underlying substrate, without requiring any post-treatment to dissolve the underlying substrate or any kind of mechanical force that could damage the membrane (see in Supplementary Figure S1A images representing the sequence of actions to detach the membrane from the PP substrate). The number of cycles has a direct influence on the thickness of the membranes and, indirectly, on their robustness and easiness to handle [50]. These membranes were designed as a support material for cells to adhere and differentiate, allowing bone tissue regeneration; this means that we needed the smallest thickness possible, without compromising the stability in physiological medium and the handling. The LbL parameters were optimized, choosing for instance a different number of cycles, but 200 bilayers seemed to be the best compromise between thickness and stability/handling.

The build-up of such LbL-based freestanding membranes has been reported in the literature [50,51], and even with polymers with a potentially adhesive character it was possible to detach the membranes; this may be due to the first layer being CHT. The photograph of both [CHT/HA]_200_ and [CHT/HA-DN]_200_ membranes is presented in Figure S1B, with some differences in color and transparency perceptible.

#### 3.3.1. Surface Morphology and Thickness of the Freestanding Membranes

**Figure 4:**
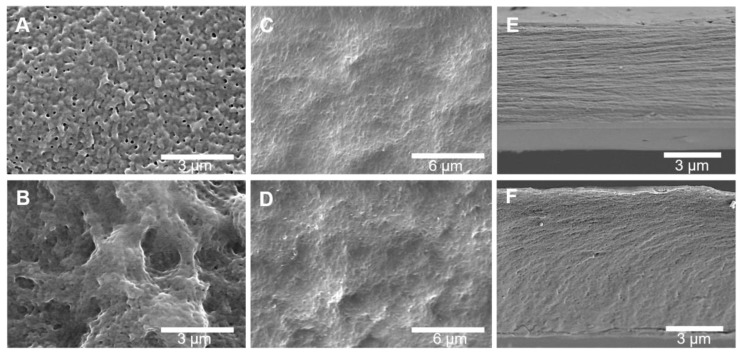
Representative scanning electron microscopy (SEM) images of the surfaces of (**A**) [CHT/HA]_200_ (HA-ending side); (**B**) [CHT/HA-DN]_200_ (HA-DN-ending side); (**C**) [CHT/HA]_200_ (CHT-ending side); (**D**) [CHT/HA-DN]_200_ (CHT-ending side). Representative SEM images of the cross-section of the (**E**) [CHT/HA]_200_ and the (**F**) [CHT/HA-DN]_200_ freestanding membranes.

The morphologies of the surface of the freestanding membranes were investigated by SEM. Figure 4A shows the top view of upper side of the [CHT/HA]_200_ membrane (HA-ending layer); a closed network pore configuration and a smooth surface was observed. In contrast, the top-view of the upper side of the [CHT/HA-DN]_200_ membrane (HA-DN-ending layer) shows an interesting pore network, with bigger pore diameters and a rougher surface, as shown in Figure 4B. Therefore, some differences were noted between the morphologies of the upper layer of the two systems; we hypothesize that the presence of DN in the last layer of the freestanding membrane conferred a higher pore network system and rougher structures than HA alone. Figure 4C,D shows a SEM image of the down side of the [CHT/HA]_200_ and [CHT/HA-DN]_200_ membranes, respectively. Similar morphologies were observed on the CHT side of the membranes, highlighting the rough nature conferred by CHT. Figure 4E,F represents the cross-section of the [CHT/HA]_200_ and [CHT/HA-DN]_200_ freestanding membranes after free-fracture, respectively; [CHT/HA]_200_ cross-section shows a more homogeneous distribution of the polyelectrolytes layers along with the thickness of the membrane, being possible to observe a kind of LbL stratification. The thickness of the [CHT/HA]_200_ membrane is around 5.5 ± 0.1 µm. A thickness of 7.7 ± 0.1 µm was achieved when DN is conjugated with HA and integrated in a multilayer system with CHT. The differences between the thickness of the membranes and the organization along the *z*-axis could be due to the arrangement of the polymer chains during the multilayer construction. Comparing the real thickness values with the ones estimated from QCM-D data, we obtained thicker [CHT/HA-DN]_200_ films than expected. This fact could be due to some accumulation phenomenon and chain arrangement along with the thickness, which could happen after the construction of the initial layers of the multilayer. Curiously, the thickness of the [CHT/HA]_200_ membranes was much lower than expected by the exponential growth. We hypothesize that for a higher number of bilayers, we cannot assume exponential growth but instead linear growth (*R*^2^ = 0.969); therefore, the estimated thickness would be about 4.4 µm, which is approximated to be the real thickness of the freestanding [CHT/HA]_200_ membrane.

#### 3.3.2. Chemical Analysis of the Surface of the Freestanding Membranes

**Figure 5:**
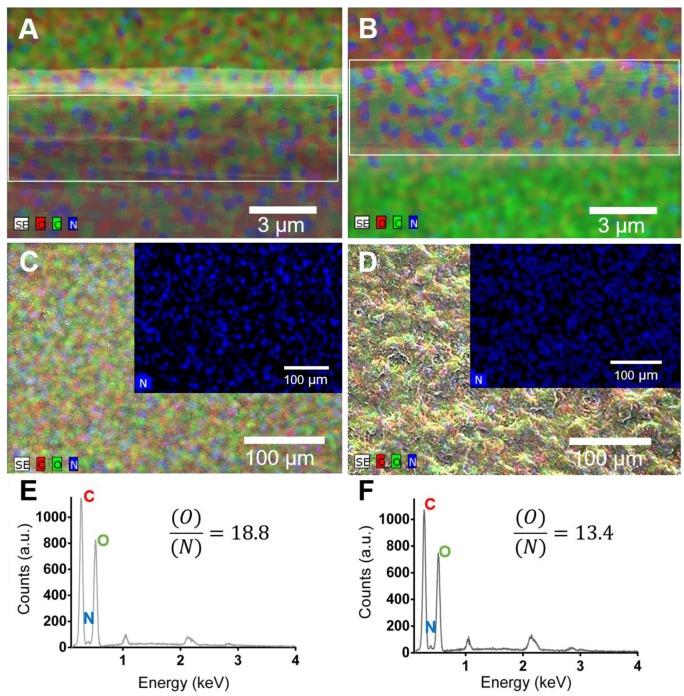
Mixed element map for carbon (C), oxygen (O) and nitrogen (N) of the cross-section of (**A**) [CHT/HA]_200_ and (**B**) [CHT/HA-DN]_200_ freestanding membranes. Mixed element map for carbon (C), oxygen (O), and nitrogen (N) of upper surface of (**C**) [CHT/HA]_200_ and (**D**) [CHT/HA-DN]_200_ freestanding membranes. Energy-dispersive X-ray spectra and ration quantification of O/N of (**E**) [CHT/HA]_200_ and (**F**) [CHT/HA-DN]_200_ freestanding membranes. a.u.: Arbitrary units.

The surface chemical properties of the freestanding multilayer membranes were investigated by EDS analysis. Figure 5A,B shows EDS maps for [CHT/HA]_200_ and [CHT/HA-DN]_200_ membranes along the thickness (cross-section). Visually, both membranes revealed the presence of carbon, oxygen and nitrogen. All these elements are presented along with all the thickness of the membranes. In turn, Figure 5C,D shows EDS map images for the [CHT/HA]_200_ and [CHT/HA-DN]_200_ membranes’ top surface and the respective quantification for the same area (Figure 5E and 5F, respectively). Both membranes revealed the presence of carbon, oxygen, sodium, and nitrogen. In fact, the ration between oxygen and nitrogen is significant higher for [CHT/HA-DN]_200_ than for [CHT/HA]_200_ membranes. We hypothesize that the nitrogen quantity could be higher for the [CHT/HA-DN]_200_ due to the presence of DN in the last layer, which adds amine groups to the system.

#### 3.3.3. Adhesive Properties of the Freestanding Membranes

**Figure 6:**
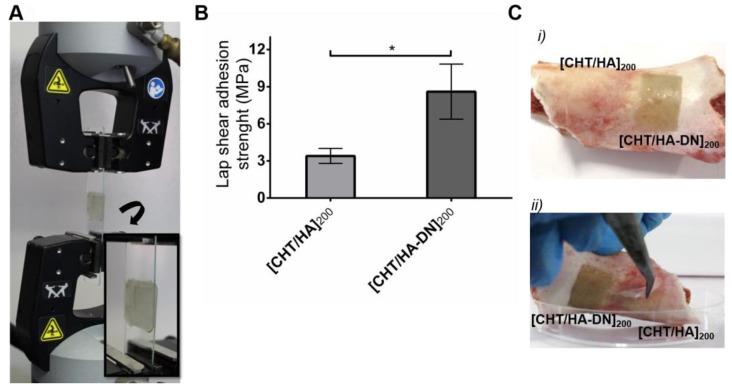
Adhesive properties of the freestanding membranes. (**A**) Mounting scheme for testing the lap shear adhesion strength on the Instron equipment; (**B**) lap shear adhesions strength values for each system. Significant differences were found for *p* < 0.05. (**C**) Representative images of the adhesiveness potential of [CHT/HA]_200_ and [CHT/HA-DN]_200_ freestanding membranes on a clean surface of porcine bone: (i) before and (ii) after applying a detachment force with tweezers.

In this work, freestanding membranes were used to glue two pieces of glass and this system was used as a proof of the concept for the adhesive strength of the [CHT/HA]_200_ and [CHT/HA-DN]_200_ membranes. The size of these membranes was around 20 mm × 20 mm, and the thickness was between 5 and 8 µm. Briefly, the freestanding membrane was sandwiched between two pieces of glass, in a controlled environment, trying to mimic the inner body conditions (37 °C in a moist environment), and incubated overnight. Most of the methods to determine the adhesive properties involve determination of the perpendicular force required to separate two surfaces. As biological tissues are more susceptible to shear stress than tensile stress, we decided to investigate the force that makes an adhesive slide on a surface in the direction parallel to the plane of contact [52]. The ability of [CHT/HA]_200_ and [CHT/HA-DN]_200_ to glue two glass substrates together was evaluated using a universal mechanical testing machine according to the standard procedure ASTM D1002, with subtle modifications. A heavy load (1.0 kN) could be held on a small joint area (20 mm × 20 mm) until it caused the detachment of the glass slides (see the mounting scheme in Figure 6A). Figure 6B presents the values of adhesive strength for each condition. From the lap shear adhesion tests, it can be seen that there were higher load values for the same displacement for [CHT/HA-DN]_200_ membranes, indicating that more load is required to separate the glued glass slides. The calculation of lap shear adhesion strength was performed for each case; the [CHT/HA]_200_ system presents an adhesion strength of 3.4 ± 0.6 MPa, while the [CHT/HA-DN]_200_ system presents an adhesion strength of 8.6 ± 2.2 MPa. Such a difference highlights the adhesive strength of the membrane containing DN compared with the control. We hypothesize that the presence of the catechol moieties in the [CHT/HA-DN]_200_ membranes increased the adhesion force between the two glass slides. The adhesive characteristics of catechol-based materials have been already investigated [17,53,54]. For instance, Ninan et al. [55] studied the adhesive properties of marine mussel adhesive extracts to bond porcine skin in controlled dry and humid conditions; the tissue joint strength was about 1 MPa for mussel extract joints under humid conditions. Also, Kim et al. [56] reported the development of a water-immiscible mussel protein-based adhesive, composed of a complex coacervate of HA and DOPA with strong underwater adhesion; an adhesive strength of about 0.14 ± 0.03 MPa was obtained, using rat bladder tissue as the contact substrate. Even using a different contact substrate, the guidelines for adhesive tests were the same as for the previous examples. Even so, we obtained significantly higher values of strength adhesion with [CHT/HA-DN]_200_ membranes. Well-known bioadhesives are fibrin and cyanoacrylate-based materials, which present a shear adhesive strength around 0.013 MPa and 0.068 MPa, respectively [57]. Bioadhesive hydrogels have been reported mainly for topical wound dressings and sealants as their adhesive strength is still considered to be weak [57]. For tissue engineering applications, bioadhesive films are of more interest. Layer-by-layer technology has been used for this purpose; Neto et al. [17,58] already reported the ability to produce multilayer coatings composed of CHT and DOPA-modified HA with enhanced adhesive properties. The adhesive strength of the coating was about 2.3 ± 2.2 MPa, more than triple that of the control coating. Other LbL systems have been reported, but mostly in the form of coatings and not as freestanding membranes, which is more relevant for developing supports for tissue engineering purposes. To the best of our knowledge, there has already been one work on such adhesive freestanding multilayer membranes, but instead of dopamine they took advantage of the adhesive properties of levan derivatives [52]. Another strategy was envisaged to evaluate the bioadhesiveness of this membrane. After putting the [CHT/HA]_200_ and [CHT/HA-DN]_200_ membranes in contact with a clean surface of porcine bone at 37 °C, at 50% humidity overnight, it was possible to observe that more force is required to pull out the [CHT/HA-DN]_200_ membrane (see representative images in Figure 6C (i) before and (ii) after applying a detachment force with tweezers, and the video recording in Supplementary Video S1). The [CHT/HA]_200_ membrane was easier to detach from the surface of porcine bone than the [CHT/HA-DN]_200_ membrane. This result is in accordance with the lap shear adhesion strength test, highlighting the adhesive potential of DN when incorporated in these multilayer freestanding systems.

### 3.4. In Vitro Cell Studies

MC3T3-E1 cells were seeded above [CHT/HA]_200_ and [CHT/HA-DN]_200_ freestanding membranes. The performance of such kind of adhesive substrates was evaluated for different cellular functions, namely the metabolic activity, cytotoxicity, proliferation, and morphology of MC3T3-E1. A preliminary immunofluorescent assay was performed to evaluate the osteogenic potential of these substrates. The MC3T3-E1 response for TCPS surfaces were used as reference and positive control.

#### 3.4.1. Metabolic Activity, Cytotoxicity, Proliferation, and Morphology of MC3T3-E1 Cells

**Figure 7:**
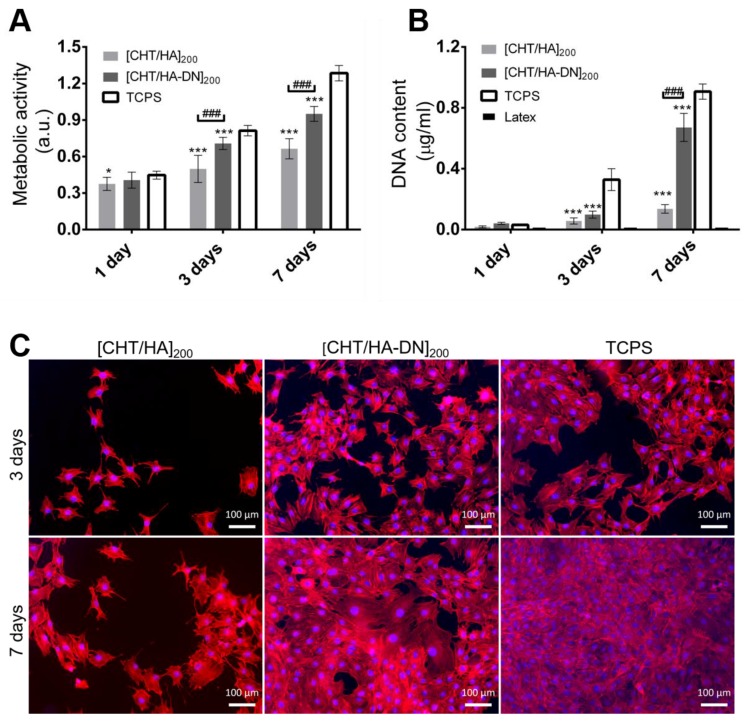
In vitro cell studies. (**A**) **Metabolic activity** of MC3T3- E1 cells seeded on the membranes (Alamar Blue assay). Significant differences were found between membranes and TCPS conditions (for * *p* < 0.05; *** *p* < 0.001) and between the two kind of systems (### *p* < 0.001); (**B**) DNA content of MC3T3- E1 seeded above the membranes (PicoGreen Kit). Significant differences were found between membranes and tissue culture polystyrene surface (TCPS) conditions (*** *p* < 0.001) and between the two kind of systems (### *p*< 0.001). (**C**) Fluorescence images of MC3T3- E1 cells stained with phalloidin (red) and 4′,6-diamidino-2-phenylindole (DAPI) (blue), at three and seven days of culture on the [CHT/HA]_200_ and [CHT/HA-DN]_200_ membranes and the TCPS (positive control). a.u.: Arbitrary units.

The metabolic activity of MC3T3-E1 cells and the cytotoxicity of [CHT/HA]_200_ and [CHT/HA-DN]_200_ membranes were evaluated by the Alamar blue colorimetric test. Figure 7A shows the results for the resulting Alamar blue absorbance. After one day of culture in basal growth medium, the cells seemed to adhere to the different substrates with no significant differences. Nevertheless, the values of absorbance of cells seeded above the freestanding membranes were comparable to the values for TCPS surfaces. This observation, combined with the increase in absorbance along with the days of culture for each condition, is an indication of the non-cytotoxicity of the materials. After three and seven days of culture, significant differences in metabolic activity were noted between [CHT/HA]_200_ and [CHT/HA-DN]_200_ freestanding membranes; higher absorbance values for cells seeded above [CHT/HA-DN]_200_ freestanding membranes indicate enhanced metabolic activity and viability. Also, the proliferation of MC3T3-E1 was estimated using a standard DNA quantification assay. Figure 7B shows the content of DNA for each condition up to seven days of culture. After one day of culture the DNA content of MC3T3-E1 cells seeded above [CHT/HA]_200_, [CHT/HA-DN]_200_, and TCPS was quite similar, while for three and seven days of culture there were significant differences between these conditions. Following the same trend observed for Alamar blue results, the highest rates of proliferation were found for [CHT/HA-DN]_200_ freestanding membranes, indicating a better cell response to the catechol-containing membranes. Additionally, the morphology of the cells was observed using a fluorescence assay (see Figure 7C). After three and seven days of culture in basal growth medium, the cells seeded above the different surfaces were fixed and stained with specific markers: phalloidin (in red) to label the actin cytoskeleton and DAPI (in blue) to label the nuclei of the cells. The representative images, presented in Figure 7C, corroborate the results of metabolic activity and DNA quantification: after three and seven days of culture, the density of cells adhered on the [CHT/HA-DN]_200_ membranes was significantly higher than for the [CHT/HA]_200_ membranes and close to the cell density on the TCPS surfaces. Moreover, the morphology of the MC3T3-E1 cells also differed; cells adhered above the [CHT/HA-DN]_200_ started to establish cell–cell contact with each other after just one day of culture, while for cells seeded on [CHT/HA]_200_ membranes this cell–cell contact was only perceptible after three days of culture; these observations reinforced the proliferation results as cell–cell contact promotes cell proliferation. We hypothesize that DN presence along the thickness of the multilayer membranes and concretely on the surface improved their biological performance at different stages: adhesion, viability, communication, and proliferation. These results could have potential applicability in the tissue engineering field, as adhesive and biocompatible substrates to support cells’ functions. There are other works [15,59,60,61] reporting the positive effect of catechol-based materials in promoting cellular adhesion and good function. Polycaprolactone scaffolds were modified using a mussel-inspired approach; polydopamine coating and hyaluronic acid immobilization seemed to be an effective way to improve cellular performance [62]. Zhang et al. [63] suggested LbL methodology to simply coat titanium implants with HA-DN and CHT, aiming to enhance the osteoblast proliferation. The greatest advantage of our system over the reported examples and the existing literature is related to the combination of a freestanding substrate composed of natural-based materials with enhanced adhesive strength and improved cell response. Moreover, this kind of flexible substrate could be produced, handled, and applied in quite a simple and adaptable way.

#### 3.4.2. Differentiation of MC3T3-E1 Cells

**Figure 8:**
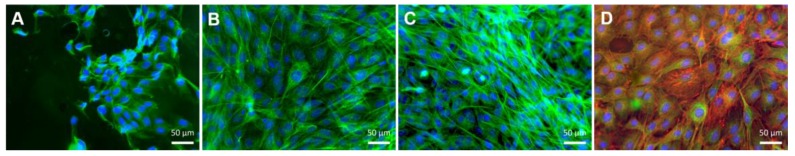
Osteopontin immunofluorescence images of MC3T3-E1 cells stained in green and with DAPI (in blue), after 14 days in osteogenic medium and cultured on the (**A**) [CHT/HA]_200_ and (**B**) [CHT/HA-DN]_200_ membranes and (**C**) TCPS (positive control); (**D**) Merged image of MC3T3-E1 cells cultured on the [CHT/HA-DN]_200_ membrane is shown in the overlay with osteopontin (green), phalloidin (red) and DAPI (blue) markers.

The differentiation of mouse MC3T3-E1 pre-osteoblasts toward the formation of a mineralized extracellular matrix was also evaluated. This cell line was chosen as the model for our system due to its usually compressed level of differentiation and the ability to form a mineralized bone-like extracellular matrix [64]. Figure 8 shows the immunofluorescence images of cells seeded on the different materials, using osteopontin as the osteogenic marker. Typical osteogenic differentiation of MC3T3-E1 cells occurs in three phases: proliferation, extracellular matrix deposition and maturation, and finally mineralization. Each phase corresponds to higher expressions of certain genes; osteopontin is expressed near the later stages of osteogenic differentiation. Therefore, after 14 days in a differentiation medium containing ascorbic acid and β-glycerolphosphate, different behaviors could be observed for the freestanding systems. The cells cultured on [CHT/HA-DN]_200_ showed stronger immunofluorescence for osteopontin protein staining than the cells cultured on [CHT/HA]_200_ membranes, and very similar fluorescence to the TCPS positive control (see Figure 8A–C). We hypothesize that catechol-based moieties provided the multilayer films with important properties to improve their osteogenic potential. The characteristic chemical groups of [CHT/HA]_200_ and [CHT/HA-DN]_200_ membranes could be considered osteogenic differentiation promoters; besides CH_2_ and CH_3_ groups, these surfaces also present NH_2_ and OH groups and for all of them a positive effect on endorsing osteogenic differentiation has been reported [65]. Few works have investigated the potential of mussel-inspired adhesive proteins for in vitro bone formation. For instance, Yu et al. [66] coated titanium substrates with polydopamine to facilitate the homogeneous covalent immobilization of collagen on their surface and promote the osteogenic differentiation of MC3T3-E1 cells. Another group [67] synthesized a conjugate of alginate and dopamine and produced alginate–dopamine gels, which seemed to promote the osteogenic differentiation of mesenchymal stem cells. The adhesive character of DN allowed the author to coat the gel with silver, providing antibacterial properties. Moreover, there are some studies reporting that the polydopamine coating could enhance hydroxyapatite nucleation and then promote mineralization; Lee et al. [68] coated 3D-printed polycaprolactone scaffolds with polydopamine to easily graft rhBMP-2. In addition, a higher amount of BMP-2 resulted in better bone tissue formation; even with small doses of this protein, they could induce osteogenic differentiation. In contrast, even in the absence of any other grafted protein or growth factor, our multilayer system based on mussel-inspired catechol groups could enhance the potential to differentiate MC3T3-E1 cells into osteoblasts. Figure 8D is a merged image of the MC3T3-E1 cells cultured on the [CHT/HA-DN]_200_ membrane, overlaying osteopontin (green), phalloidin (red), and DAPI (blue) markers. As phalloidin stains the actin cytoskeleton of the cells and DAPI stains the nucleus, the appearance of osteopontin as an intracytoplasmic marker is clear from Figure 8D. We hypothesize that this positive intracellular activity could be related to the cellular calcification induced by the substrate and the culture conditions during the active stage of the differentiation of MC3T3-E1 cells in osteoblasts [69].

Note that even after 21 days immersed in a physiological medium, the membranes seemed to be stable, but presented some signs of degradation.

Overall, the [CHT/HA-DN]_200_ freestanding membranes showed enhanced adhesive strength properties, as well as improved cell adhesion, proliferation, and differentiation, making them a good candidate to regenerate bone tissue.

## 4. Conclusions

The notable ability of DOPA and its analogues to form strong interactions with both organic and inorganic surfaces was inspired by the process of wet adhesion in mussels and has been used to produce materials with unique adhesion properties. Bioadhesive materials have started to gain importance in bone tissue engineering strategies, which have been appearing to overcome some issues, often related to implant failure, by creating a system that directly promotes bone ingrowth into the material’s structure and helps with tissue regeneration. Other features have been reported as significant for the success of the bone tissue engineering system. Using the LbL technique offers a unique vehicle to create a biomimetic environment due to the ability to incorporate materials that are presented in the ECM or have a bioactive role and assemble them into a functional tissue-like unit.

In conclusion, we covalently bonded DN on the backbone of HA with success, conferring important properties to this glycosaminoglycan. Taking advantage of the conjugate HA-DN and their negative nature at pH 5.5, we produced thin multilayer freestanding membranes composed only of CHT and HA-DN, using a simple LbL technology based on electrostatic interactions. Interestingly, when comparing membranes without DN with membranes where DN was conjugated with HA, we clearly enhanced the adhesive strength. MC3T3-E1 cell adhesion, viability, proliferation, and density were enhanced when cultured on [CHT/HA-DN]_200_. We hypothesize that the mechanical and morphological differences between the different multilayer systems had a positive impact on cellular behavior. Additionally, our preliminary results for MC3T3-E1 differentiation studies suggest that the presence of HA-DN on the multilayer membranes provided better differentiation signals. We assume that enhanced differentiation of MC3T3-E1 cells is related to the morphology, chemistry, and mechanical properties conferred by this catechol-based material. Therefore, our investigation suggests a cheap, scalable, and versatile technology to produce biocompatible and osteophilic [CHT/HA-DN]_200_ multilayer membranes with interesting adhesive properties that could potentially be applied in bone regeneration.

## Supplementary Materials

The following are available online at http://www.mdpi.com/2313-7673/2/4/19/s1: Figure S1: Production of the freestanding multilayer membranes; Video S1: Adhesiveness of the freestanding membranes to a clean surface of porcine bone.
