# Supplementary material for: Cell-Adhesive Bioinspired and Catechol-Based Multilayer Freestanding Membranes for Bone Tissue Engineering
Source: Biomimetics (Basel). 2017 Oct 5;2(4):19. doi: 10.3390/biomimetics2040019 (PMC6352653; doi:10.3390/biomimetics2040019)
Supplement: Supplementary file 1 [file biomimetics-02-00019-s001.zip › biomimetics-02-00019-s001.pdf]

Biomimetics **2017**, *2*, 19

Supplementary Materials: Cell-Adhesive Bioinspired and Catechol-Based Multilayer Freestanding Membranes for Bone Tissue Engineering

Maria P. Sousa and João F. Mano *

CICECO—Aveiro Institute of Materials, Department of Chemistry, University of Aveiro, 3810-193 Aveiro, Portugal; mariajsousa@ua.pt

* Correspondence: jmano@ua.pt; Tel.: +351-234-370-733

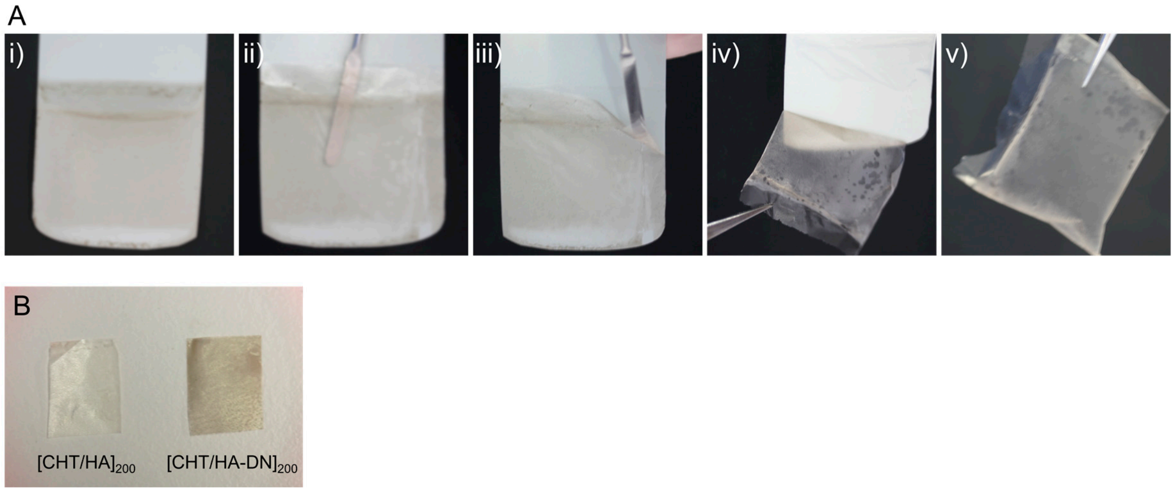

Figure S1. Production of the freestanding multilayer membranes. Images showing (**A**) the detachment process of the freestanding membranes and (**B**) both resulting $[CHT/HA]_{200}$ and $[CHT/HA\text{-}DN]_{200}$ freestanding membranes.
